# Twice upon a time: The progression of canine visceral leishmaniasis in an Argentinean city

**DOI:** 10.1371/journal.pone.0219395

**Published:** 2019-07-05

**Authors:** Daniela Lamattina, Pablo Eduardo Berrozpe, Natalia Casas, Sofía Lorian Moya, Magalí Gabriela Giuliani, Sebastián Andrés Costa, Juan Pablo Arrabal, Mariela Florencia Martínez, María Romina Rivero, Martín Salas, Cristian Alejandro Humeres, Domingo Javier Liotta, María Belén Meichtry, Oscar Daniel Salomón

**Affiliations:** 1 Instituto Nacional de Medicina Tropical (INMeT), Ministerio de Salud y Desarrollo Social de la Nación, Puerto Iguazú, Misiones, Argentina; 2 Consejo Nacional de Investigaciones Científicas y Técnicas (CONICET), Buenos Aires, Argentina; 3 Dirección Nacional de Epidemiología y Análisis de la Situación de Salud, Ministerio de Salud y Desarrollo Social de la Nación, Buenos Aires, Argentina; 4 Instituto de Biología Subtropical, Facultad de Ciencias Forestales, Universidad Nacional de Misiones, Puerto Iguazú, Misiones, Argentina; University of Minnesota, UNITED STATES

## Abstract

Canine Visceral Leishmaniasis (CVL) prevalence, spatial distribution and associated factors were assessed in four locations in Iguazú department in 2014 and in Puerto Iguazú city again in 2018. The city areas were divided into a grid of 400x400m cells. All cells were sampled in 2014 and a random subsampling was developed in 2018. In each cell, five dogs clustered in a ‘critical scenario’ (prone to have vectors) were sampled. A rapid immunochromatographic dipstick was used to detect antibodies against *Leishmania infantum*, confirming by lymph node smears observation and PCR. For Puerto Iguazú, Generalized Linear Models (GLMs) were constructed considering environmental, dog and clinical variables. Pearson's Chi square and Fisher's exact tests were employed to evaluate the association between CVL, dog clinical signs and infestation with other parasites. Cartographic outputs were made and Moran's I indices were calculated as spatial autocorrelation indicators. CVL prevalence rates were 26.18% in 2014 and 17.50% in 2018. No associations were established in environmental models, but dog age and repellent use were significant when running 2014 dog models. Clinical models showed significant associations between seropositive dogs and ophthalmological, dermal signs and onychogryphosis in 2014. In 2018, only adenomegaly was associated. The results of global Moran´s I were not significant but regarding local analysis, six sites in 2014 and one in 2018 presented autocorrelation with neighboring sites. The decrease in CVL prevalence may be associated to transmission stabilization, which could explain the lack of associations with dog-related variables. Further, spatial distribution of CVL is a poor evidence for design of transmission control measures but could be important in case of intensive parasite circulation or when the first autochthonous cases appear. For control success, sensitivity of diagnostic methods, political will and adequate material resources remain critical. Modeling of multiple variables will be required to identify factors that drive disease stabilization/destabilization.

## Introduction

Domestic dogs are the main urban reservoir of *Leishmania infantum* (Kinetoplastida: Trypanosomatidae), causative agent of human (HVL) and canine visceral leishmaniasis (CVL) in the Americas [1,2]. The protozoan is transmitted to a susceptible host through the bite of phlebotomine sandflies (Diptera: Psychodidae: Phlebotominae), and between canines also by vertical and venereal transmission [2–4].

The occurrence of HVL cases tends to cluster close to areas with a higher incidence of CVL [5,6]. Multiple factors such as dog size, age and presence of ectoparasites have been found to be associated with CVL seroprevalence [7,8], highlighting the necessity to address CVL epidemiology.

In Argentina, after the spread of CVL and HVL urban transmission from northeastern Brazil during the last decades of the past century [9], *Lutzomyia longipalpis* the main vector of *L*. *infantum* was recorded for the first time in urban landscapes in 2004 on the border with Paraguay [10]. The first autochthonous case of HVL in an Argentinean city concomitant with CVL cases were reported in 2006, also on the border with Paraguay, but 300 km away from the former focus [11]. In 2010, CVL vector and cases were found in Puerto Iguazú on the three-country border with Paraguay and Brazil, 300 km from the other two foci, as in an apex of an equilateral triangle [12], and CVL and HVL were reported since 2013–2014 [13]. Cases of HVL in the Paraguayan side of the border were known previously since 2008 [14], and the vector in the Brazilian side appeared in 2012 [15]. Given this scenario a multidisciplinary study in mirror-cities of the three countries started in 2014. The possible ways of dispersion and longitudinal changes in the distribution of vectors during these initial periods were also studied in other cities of the region [16–19]. Despite the expected, cases of HVL tend to decrease in older foci in Argentina [20] without systematic control interventions that could explain this pattern [21,22]. In turn, little is known about CVL changes in prevalence patterns during the same period. Understanding the processes associated with the recent urban emergence and progression of urban visceral leishmaniasis in the southern region of the Americas will contribute to develop control strategies. Therefore, in order to shed light into CVL progression in northeastern Argentina, CVL prevalence, spatial distribution and factors associated with infection and clinical presentation were assessed in Puerto Iguazú in 2014 and 2018. These analyses provide relevant information not only at epidemiological level but also to improve integrative public health control approaches.

## Materials and methods

Study area and study design: The study was carried out in the Iguazú department, northwestern Misiones Province, Argentina. This area borders the neighboring countries of Brazil to the north and Paraguay to the west. In addition, the area is well known for receiving more than 1 million tourists from around the world that visit the Iguazú National Park each year.

**Fig 1:**
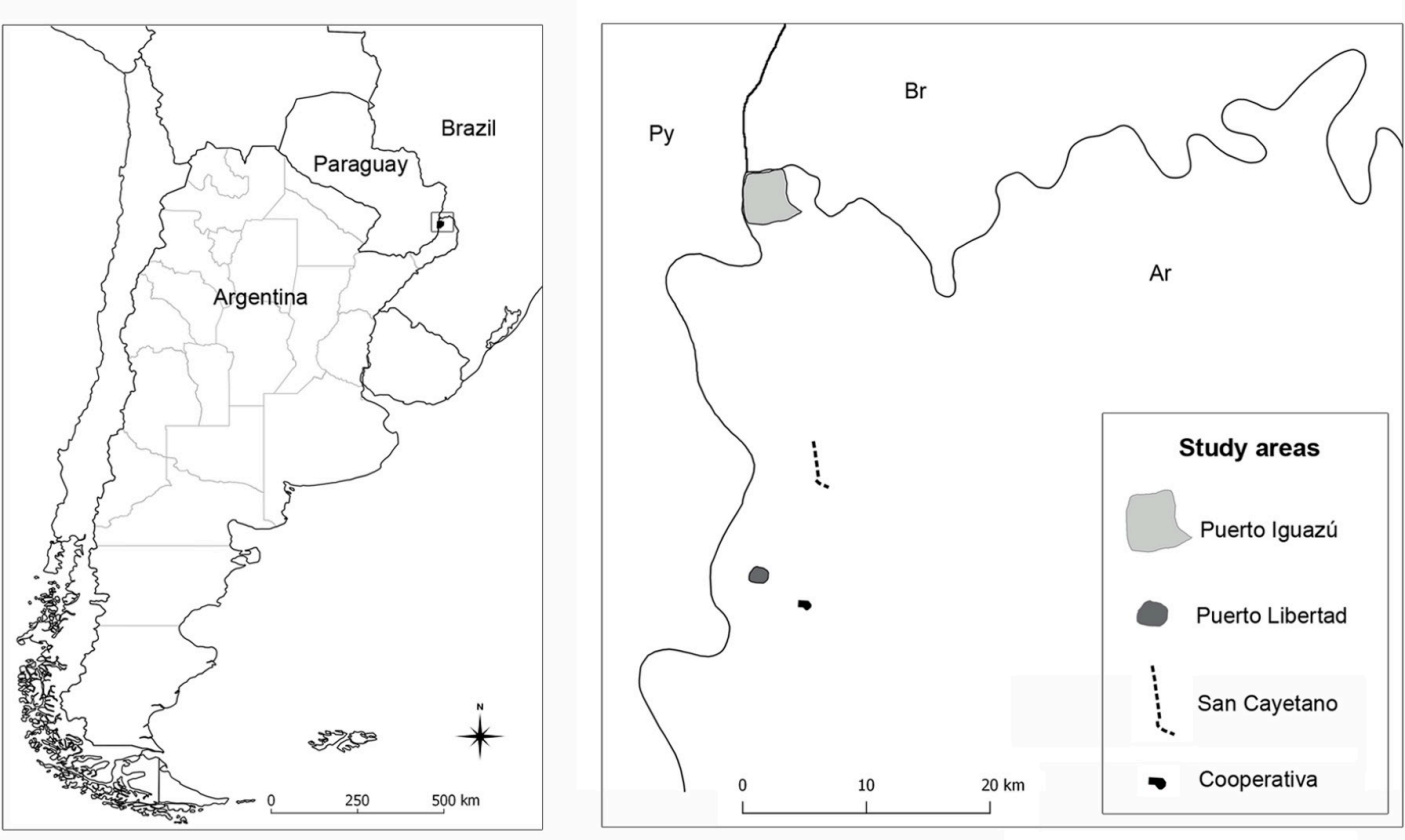
Study areas location in the three-country border of Argentina, Brazil and Paraguay, Misiones Province, northeastern Argentina. Figure made with QGIS v.2.18.15 using data from National Geographic Institute, available in: http://www.ign.gob.ar/NuestrasActividades/InformacionGeoespacial/CapasSIG (downloaded on April 17, 2018).

The study includes the cities of Puerto Iguazú (PI) (25°36'56"S, 54°34'27"W) and Puerto Libertad (PL) (25°55'11"S, 54°35'4"W) (Fig 1) that were divided into a grid of 400x400m cells. In each cell of the grid, a domicile was selected taking into account previously defined environmental features of a ‘critical scenario’ for phlebotomine proliferation and therefore, for higher probability of *L*. *infantum* transmission: high humidity, shadow, high proportion of organic matter on the soil from fruit trees and/or animal feces, blood sources as hens and dogs [23,24]. In addition, two areas of low human population density with rural-forest features were selected to compare different landscapes: the "Cooperativa" (CP) neighborhood (25°56'38"S, 54°32'40"W), located 2km north of Wanda city, and the "San Cayetano" (SC) neighborhood (25°50'10"S, 54°32'6"W), adjacent to the Urugua-Í lake, both areas surrounded by remnant Paranaense forest patches (Fig 1). All sampling sites were georeferenced using a handheld eTrex 30 global positioning system unit (Garmin International Inc., Olathe, Kansas, USA).

Two CVL sampling sessions took place in December 2014 and July 2018, respectively. The first one covered 56 cells in PI (all cells of the PI grid) and 15 in PL. In CP, eight domiciles were selected, and SC included 11 domiciles. The second sampling session included 32 cells (random cell sub-sampling) only in PI due to the low prevalence detected in the other surveyed locations. Although the total amount of sites sampled in 2018 in PI was 43% lower that the sites in 2014 due to operational restrictions, the total number of sampled dogs was four times higher than the minimum sample size needed for an expected equivalent prevalence (n_0_ = 41, CI 90%). The sites and dogs between the two sampling sessions were different for prevalence comparative purposes since the 2014 visit to a house could generated individual practices that biased the results of the 2018 visit as we would expect to assess in a cohort study design.

### Dog sampling

In each selected domicile, five CVL-unvaccinated dogs older than six months were sampled, regardless of their clinical status. In case of less than five dogs were found in the selected domicile of the quadrant, randomly selected dogs were sampled in adjacent dwellings in the same sector of the grid. All sampled dogs had owners and all dog owners gave written consent to use these samples in the study.

Whole blood was collected in 8ml plastic tubes from the cephalic vein in order to perform a serological rapid test. Additionally, a fine needle aspiration (FNA) was done in dogs tested positive to sample prescapular or popliteal lymph nodes for confirmation of serological tests. One part of the FNA was fixed on glass slides and the rest was collected in 1.5ml tubes with 300μl of phosphate-buffered saline solution (PBS). Samples were transported to the National Institute of Tropical Medicine (INMeT) for processing.

A complete physical examination of the dogs was performed by veterinarians looking for clinical signs of CVL and a survey was conducted to dog owners, by which data on dog characteristics and habits and environmental features were collected. Descriptions on the data gathered are shown in Table 1.

**Table 1:** Variables collected during fieldwork by dog physical examination and surveys conducted to dog owners from Iguazú department, Misiones province, in years 2014 and 2018.

Variable type	Variable	Description
Environmental variables	Number of dogs	Number of dogs in the house
	Number of hens	Number of hens within the property boundaries
	Fruit trees	Presence / Absence of fruit trees
	Street material	Paved / Unpaved street
Dog variables	Dog size	Small: less than 10kg
		Medium: 10 to 25kg
		Large: more than 25kg
	Dog sex	Male / Female
	Dog breed	Purebred / Crossbred
	Dog age	Pup: 6 to 12 months old.
		Adult: 1 to 10 years old
		Old: more than 10 years old
	Dog resting place	Dog sleeps outside the house
		Dog sleeps inside the house
	Dog neutering	Neutered / Not neutered
	Dog provenance	Dog was borne in the same neighborhood
		Dog was borne in another neighborhood
	Dog habitat limits	Dog freely leaves owners property
		Dog does not leave owners property
	Dog repellent	Owner applies pyrethroid repellent to the dog
		Owner does not apply pyrethroid repellent to the dog
	Ectoparasites	Presence / Absence of ticks, fleas, lice
Clinical signs	Dog body condition	Good: iliac crest bones, vertebrae and ribs not visible
		Regular: iliac crest bones, vertebrae and ribs visible
		Bad: iliac crest bones, vertebrae and ribs visible and pronounced
	Dog attitude	Active / Apathetic / Lethargic
	Ophtalmological signs	Blepharitis / Uveitis / Eye secretions
		Conjunctivitis / Keratitis / None
	Dermal signs	Erythema / Pruritus / Ulcers / Nodules
		Localized alopecia / Generalized alopecia
		Hyperkeratosis / Seborrhea / None
	Mucosal signs	Pallor / Epistaxis / Ulcers
		Nodules / None
	Adenomegaly	Presence / Absence of enlarged lymph nodes
		Number and location of affected lymph nodes
	Onychogryphosis	Presence / Absence of increased nail growth

In addition, in 2014 canine fecal samples were collected from the environment at 48 sites in PI, seven sites in PL, six in SC and four in CP (65 sites in total) during the first sampling session, for parasitological analysis. In the same sampling session, additional blood samples from 172 dogs at 52 sites in PI, 51 dogs at 15 sites in PL, 22 dogs at nine sites in SC and 20 dogs at eight sites in CP were collected in 1.5ml tubes with EDTA for microfilariae analysis.

All procedures were performed with the endorsement of the Clinical Investigation Ethics Committee (CEIC Dr. Barclay), revision 1108/26/2014, under the IDRC-Canada project #107577–001.

### Laboratory analysis

To detect circulating antibodies against *L*. *infantum* rK39 recombinant protein in fresh dog sera, we used a rapid immunochromatographic dipstick assay (Kalazar Detect canine rapid test, InBiOS International, Seattle, USA) (RDT) following the manufacturer’s instructions. Lymph node smears were fixed with methanol and stained with Giemsa for *Leishmania* sp. parasite detection by microscopy. Lymph node samples with PBS were subjected to Polymerase Chain Reaction (PCR) targeting *Leishmania* ribosomal internal transcribed spacer (*ITS-1*), which has an expected 300-350bp product [25]. The WHO *Leishmania braziliensis* MHOM/BR/75/M2903 reference strain was used as positive control. After purification, positive PCR products were sequenced and each sequence quality was evaluated and analyzed using Codon Code aligner software v 3.0.1 (CodonCode Corporation, Dedham, Massachusetts, USA). *Leishmania infantum* identity was confirmed by Blast Nucleotide Standard software (blast.ncbi.nlm.nih.gov/Blast).

Fecal samples were processed by flotation with Benbrook solution (saturated sugar solution) and by sedimentation using modified Telemann method (formaldehyde-NaCl solution and ether) to concentrate helminth eggs and protozoan cysts and oocysts [26]. In addition, samples were stained using Kinyoun technique to detect the presence of protozoan oocysts [27]. In turn, additional blood samples collected from 265 dogs were processed with the modified Knott concentration technique to detect the presence of microfilariae stained with blue methylene by optical microscopy [28].

### Statistical analysis

Prevalence was compared among sampling areas using exact Fisher´s test. PI was selected to analyze associations between CVL infection and different explanatory variables since the PI prevalence rate was significantly higher than that of the other zones (lower than 10%). Difference of prevalence rates between sampling years was assessed by Pearson's Chi square and Tukey´s contrast tests. Taking into account the results, statistical analyses were carried out for each sampling year separately.

Using R software version 3.5.1 [29], three sets of Generalized Linear Models (GLMs) with logit link were constructed for each sampling year considering environmental (number of dogs, number of hens, fruit trees and street material), dog (dog size, breed, sex, age, neutering, resting place, habitat limits, repellent, ectoparasites), and clinical variables (dog body condition, attitude, presence of ophthalmological signs, dermal signs, mucosal signs, adenomegaly, onychogryphosis). These models were simplified by a stepwise removing of terms according to the Akaike´s Information Criterion (AIC) retaining only the variables that decreased the model's AIC by at least two units. Models with the lowest AIC were considered the best models of the sets [30].

Multicollinearity between explanatory variables was assessed by calculating the Variance Inflation Factors (VIF) in global models, using a cut-off value of 3 to remove collinear variables [31]. The inclusion of sampling sites as a random effect was discarded by comparing a Generalized Linear Mixed Model (GLMM) using lme4 package with a model fitted just with fixed effects. When required, Tukey´s contrast tests were used to compare levels of categorical variables.

Pearson's Chi square independence test and Fisher's exact test were employed to evaluate the association between CVL infection, dog clinical signs and infestation with other parasites. Odds ratios of associated variables were calculated.

### Spatial analysis

QGIS v.2.18.15 [32] cartographic outputs were used to characterize prevalence spatial distribution by Inverse Distance Weighted (IDW) interpolation and to identify sites with spatial dependence.

The global and local Moran's I indices were calculated as indicators of spatial autocorrelation of CVL prevalence rates for the two sampling periods. Global Moran´s I evaluates differences between the assessed distribution and a random distribution pattern of the cases in the study area. Local Moran's I denotes the existence of autocorrelation between the prevalence values of a sampling site and the values of neighboring sites and classifies them according to their location in one of four quadrants of Moran´s Scatterplot: i) H/H indicates sites with positive spatial correlation (spatial clusters) that are spatial units with standardized values above the mean which, in turn, have neighbors with high standardized values, determining hot spots, ii) H/L indicates a negative spatial correlation between spatial units of high standardized values and neighbors of low values, iii) L/L indicates sites with positive spatial correlation but spatial units and their neighbors have standardized values below the mean, determining cold spots, iv) L/H identifies points of standardized values lower than the mean that present negative spatial correlation relative to neighbors with standardized values higher than the mean [33].

## Results

**Fig 2:**
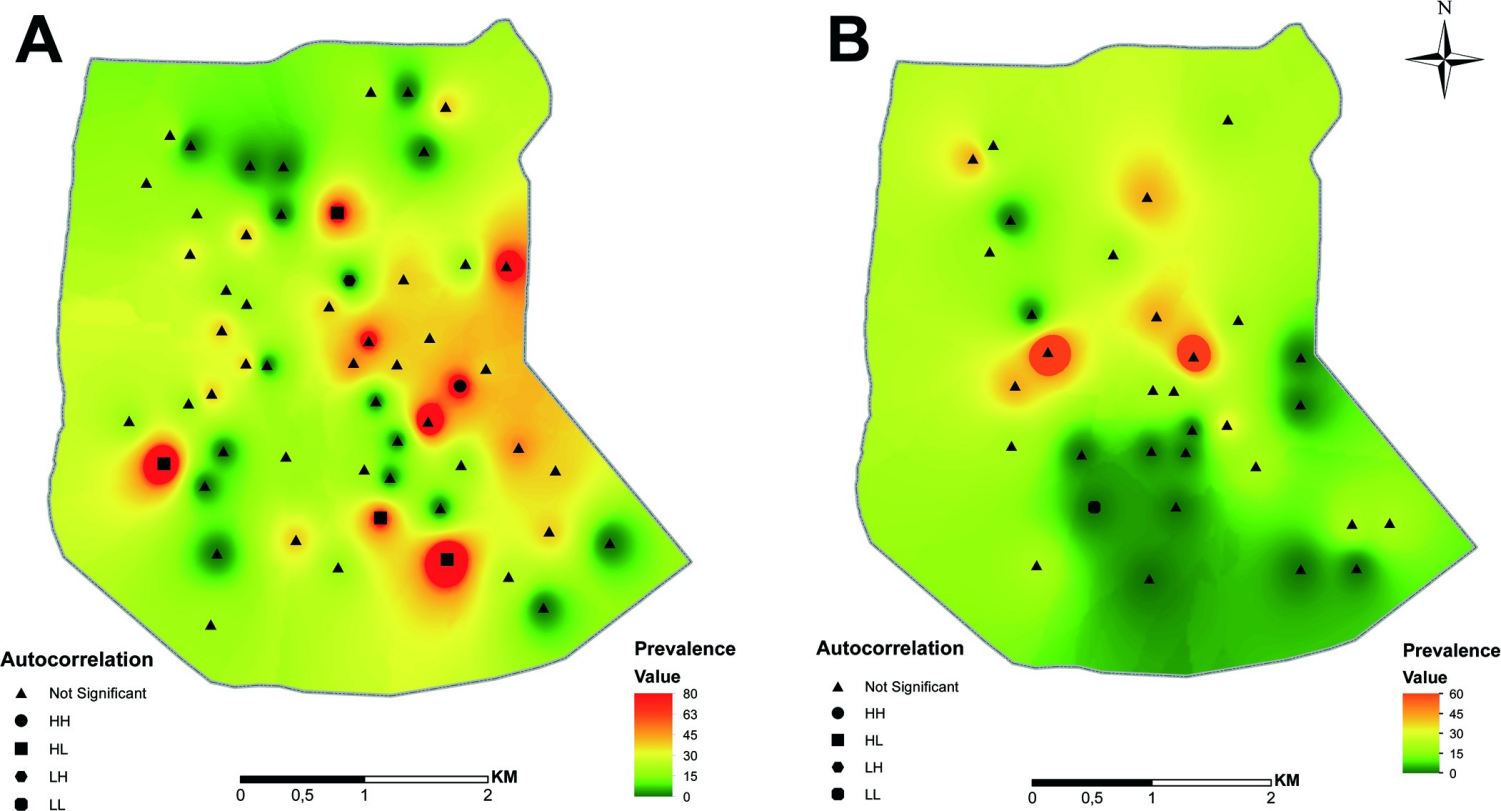
**Distribution, prevalence values and Moran´s scatterplot autocorrelation identities of Puerto Iguazú sampling sites A. Year 2014. B. Year 2018.** Figure made by the authors with QGIS v.2.18.15.

A total of 405 samples were collected from dogs in PI (n = 275), PL (n = 74), CP (n = 25) and SC (n = 31) in 2014. The overall seropositivity by RDT was 19.06%, but significant differences were found between sampling zones. Prevalence in PL (2.70%), SC (9.68%), CP (0%) and PI (26.18%) were significantly different (F = 34.509, p<0.001), with prevalence per site ranging from 0 to 80% (Fig 2A). Prevalence in PL was significantly lower than in PI (z = -3.49, p = 0.002). Taking into account the high prevalence in this city, only data collected in PI were employed to search for spatial patterns and variable associations in the models. In 2018, 160 dogs were sampled in PI, of which 28 (17.50%) were positive to the serological test, with prevalence per site ranging from 0 to 60% (Fig 2B). Differences in prevalence between sampling years 2014 and 2018 was significant, being lower in year 2018 (z = -2.063, p = 0.039). Results of all collected variables can be seen in Table 2.

**Table 2:** Results of collected data on environmental, dog and clinical signs variables of dogs from Puerto Iguazú (PI), Puerto Libertad (PL), San Cayetano (SC) and Cooperativa (CP) in years 2014 and 2018.

Year		2014	2014	2014	2014	2018
Area		PI n (%)	PL n (%)	SC n (%)	CP n (%)	PI n (%)
Dog size	Small	108 (39.3)	19 (25.7)	10 (32.3)	10 (40)	26 (16.3)
	Medium	109 (39.6)	41 (55.4)	15 (48.4)	10 (40)	113 (70.6)
	Large	57 (20.7)	13 (17.6)	6 (19.4)	5 (20)	19 (11.9)
	N/D	1 (0.4)	1 (1.4)	-	-	2 (1.2)
Dog sex	Female	132 (48)	25 (33.8)	14 (45.2)	12 (48)	83 (51.9)
	Male	141 (51.3)	49 (66.2)	17 (54.8)	13 (52)	77 (48.1)
	N/D	2 (0.7)	-	-	-	-
Dog breed	Purebred	55 (20)	7 (9.5)	3 (9.7)	-	16 (10)
	Crossbred	219 (79.6)	65 (87.8)	28 (90.3)	25 (100)	144 (90)
	N/D	1 (0.4)	2 (2.7)	-	-	-
Dog body condition	Good	229 (83.3)	66 (89.2)	25 (80.6)	18 (72)	147 (91.9)
	Regular	34 (12.4)	6 (8.1)	5 (16.1)	6 (24)	13 (8.1)
	Bad	8 (2.9)	2 (2.7)	1 (3.2)	1 (4)	-
	N/D	4 (1.4)	-	-	-	-
Dog attitude	Active	235 (85.5)	73 (98.6)	27 (87.1)	25 (100)	158 (98.8)
	Apathetic	10 (3.6)	1 (1.4)	3 (9.7)	-	2 (1.2)
	Lethargic	6 (2.2)	-	1 (3.2)	-	-
	N/D	24 (8.7)	-	-	-	-
Ophtalmological signs	Blepharitis	5 (1.8)	-	-	-	8 (5)
	Uveitis	1 (0.4)	-	-	-	-
	Eye secretions	25 (9.1)	9 (12.2)	5 (16.1)	-	23 (14.4)
	Conjunctivitis	19 (6.9)	-	1 (3.2)	-	5 (3.1)
	Keratitis	3 (1.1)	-	2 (6.5)	-	3 (1.9)
	None	228 (82.9)	64 (86.5)	24 (77.4)	25 (100)	135 (84.4)
	N/D	6 (2.2)	1 (1.4)	-	-	-
Dermal signs	Erythema	-	-	-	-	1 (0.6)
	Pruritus	4 (1.4)	-	-	-	1 (0.6)
	Ulcers	37 (13.4)	1 (1.4)	1 (3.2)	-	27 (16.9)
	Nodules	-	-	-	-	2 (1.2)
	Localized alopecia	40 (14.5)	6 (8.1)	6 (19.4)	-	61 (38.1)
	Generalized alopecia	5 (1.8)	5 (6.8)	5 (16.1)	-	4 (2.5)
	Hyperkeratosis	6 (2.2)	-	-	-	18 (11.3)
	Seborrhea	6 (2.2)	3 (4.1)	3 (9.7)	4 (16)	15 (9.4)
	None	190 (69.1)	61 (82.4)	19 (61.3)	21 (84)	83 (51.9)
	N/D	9 (3.3)	1 (1.4)	-	-	-
Adenomegaly	Yes	174 (63.3)	27 (36.5)	19 (61.3)	7 (28)	69 (43.1)
	No	86 (31.3)	45 (60.8)	12 (38.7)	18 (72)	91 (56.9)
	N/D	15 (5.4)	2 (2.7)	-	-	-
Onycogryphosis	Yes	49 (17.8)	5 (6.8)	11 (35.5)	3 (12)	10 (6.3)
	No	214 (77.8)	67 (90.5)	20 (64.5)	22 (88)	150 (93.7)
	N/D	12 (4.4)	2 (2.7)	-	-	-
Ectoparasites	Yes	143 (52)	35 (47.3)	19 (61.3)	9 (36)	118 (73.8)
	No	125 (45.5)	34 (45.9)	12 (38.7)	16 (64)	42 (26.2)
	N/D	7 (2.5)	5 (6.8)	-	-	-
Dog age	Pup	36 (13.1)	15 (20.3)	3 (9.7)	2 (8)	8 (5)
	Adult	222 (80.7)	55 (74.3)	28 (90.3)	23 (92)	135 (84.4)
	Old	17 (6.2)	4 (5.4)	-	-	17 (10.6)
Dog resting place	Inside	22 (8)	71 (95.9)	-	1 (4)	9 (5.6)
	Outside	252 (91.6)	3 (4.1)	31 (100)	24 (96)	151 (94.4)
	N/D	1 (0.4)	-	-	-	-
Dog neutering	Neutered	26 (9.4)	4 (5.4)	-	-	23 (14.4)
	Not neutered	248 (90.2)	70 (94.6)	31 (100)	25 (100)	137 (85.6)
	N/D	1 (0.4)	-	-	-	-
Dog provenance	Same neighborhood	136 (49.4)	34 (45.9)	14 (45.2)	18 (72)	72 (45)
	Other neighborhood	131 (47.6)	33 (44.6)	12 (38.7)	7 (28)	86 (53.8)
	N/D	8 (2.9)	7 (9.5)	5 (16.1)	-	2 (1.2)
Dog habitat limits	Leaves property	191 (69.5)	57 (77)	25 (80.6)	16 (64)	112 (70)
	Does not leave	82 (29.8)	16 (21.6)	6 (19.4)	9 (36)	48 (30)
	N/D	2 (0.7)	1 (1.4)	-	-	-
Dog repellent	Applies	32 (11.6)	16 (21.6)	-	-	14 (8.8)
	Does not apply	233 (84.7)	48 (64.9)	31 (100)	25 (100)	146 (91.2)
	N/D	10 (3.6)	10 (13.5)	-	-	-
Total number of dogs		275	74	31	25	160

In 2014, a total of 57 lymph node smears were positive (77% of the positive dogs) and 52 (70.3%) lymph node samples showed positive PCR products. *ITS-1* sequences shared >99% identity with a *L*. *infantum* sequence available in GenBank (KM677134.1). All sequences were deposited at GenBank and assigned accession numbers MK160162 to MK165213. In 2018, 14 lymph node smears were positive (50% of seropositive dogs) and 21 lymph node samples were positive to *Leishmania* sp. *ITS-1* amplicons (75% of seropositive dogs).

Among dogs infested with ectoparasites in 2014, 122 had *Rhipicephalus sanguineus* sensu lato (30.1%) and 129 had *Ctenocephalides felis* (31.9%). In 2018, 55 dogs had *R*. *sanguineus* s.l. (34.4%) and 105 were infested with *C*. *felis* (65.6%).

Among the 65 sites where fecal samples were collected, 45 (69.2%) resulted positive for the presence of parasitic helminth eggs, one of which was positive for *Toxocara canis* eggs, five for eggs of *Trichuris vulpis* and 28 to Strongylid eggs, while nine sites were positive for the presence of both Strongylid and *T*. *canis* eggs, and two sites were positive for the presence of Strongylid and *T*. *vulpis* eggs. The presence of protozoa was detected in nine sites (13.9%): four with *Giardia lamblia*, four with *Isospora canis* and one with *Eimeria* sp. On the other hand, laboratory tests revealed 12 dogs (4.5%) positive to microfilariae of the Onchocercidae family, of which two had *Acanthocheilonema reconditum*, seven had *Dirofilaria immitis* and three had both filarial species.

Values of VIFs were lower than 3, therefore no collinearity was found among variables.

After stepwise elimination of terms, no significant associations were established in the models with environmental variables between positive dogs and the number of dogs and hens, presence of fruit trees or street type and their interactions. In turn, dog age and dog repellent were significant when running 2014 GLMs with dog variables. The AIC of the model containing these two variables was 304.5, and the effect of removing one of the variables was an AIC increment of 4.75. The ΔAIC of this model and the null model was 9.08. The model indicated a significant difference between adult dogs and pups (p = 0.0157) and Tukey´s test confirmed the ages with significant differences were adult and pup (z = -2.416, p = 0.0395). Odds ratio of adult age was 4.508 (95% CI = 1.536–19.267), meaning adult dogs had 450% the chances of the pups to have detectable antibodies. Regarding repellent application, dogs that owners did not apply spot-on formulations or collars with pyrethroid repellent had 420% the chances of dogs with regular repellent applications to be seropositive (OR = 4.199, 95% CI = 1.389–18.331). Clinical signs models showed significant associations between seropositive dogs and presence of ophthalmological signs (OR = 2.16, 95% CI = 1.11–4.15), dermal signs (OR = 2.54, 95% CI = 1.42–4.49) and onychogryphosis (OR = 1.99, 95% CI = 1.05–3.68). The AIC of this model was 366.4, ΔAIC removing one variable was 2.41 and ΔAIC of null model was 27.47.

Among seropositive canines in 2014 (77), 18.2% were asymptomatic, while 42.9% were oligosymptomatic (presence of one or two clinical signs) and 39% had more than two clinical signs (polysymptomatic). Among seronegative dogs (328), 34.4% were asymptomatic, 50.2% were oligosymptomatic and 15.5% were polysymptomatic. A significant difference was found in the 2014 prevalence depending on the presence of clinical signs compatible with CVL (z = 23.032, p<0.001). Using Tukey´s contrast test, significant differences were found between polysymptomatic and asymptomatic dogs (z = 4.264, p<0.001) and between polysymptomatic and oligosymptomatic dogs (z = 3.605, p<0.001).

Regarding the second sampling session in PI in 2018, dog variables were found not to be associated with the detection of antibodies against *Leishmania* rK39 recombinant protein and the only clinical sign associated was the presence of adenomegaly. The AIC of the clinical signs model was 143.9 and ΔAIC of the null model was 6.45. Dogs with at least one enlarged lymph node had 350% the chances to be seropositive (OR = 3.46, 95%CI = 1.49–8.59).

Among seronegative dogs in 2018 (132), 43.2% were asymptomatic, 53% were oligosymptomatic and 3.8% were polysymptomatic, while among seropositive dogs (28), there were no asymptomatic individuals, 71.4% were oligosymptomatic and 28.6% were polysymptomatic. Fisher exact test showed a significant difference in prevalence depending on the number of CVL clinical signs (z = 32.495, p<0.001) and Tukey´s test showed a significant difference between oligosymptomatic and polysymptomatic dogs (z = 2.761, p = 0.0115).

When ectoparasites were analyzed taking into account fleas and ticks as a whole, there were no significant differences between CVL positive and negative dogs. However, *R*. *sanguineus* presence was associated with the presence of antibodies against *Leishmania* rK39 recombinant protein in 2014 (z = 4.188, p = 0.041). In 2018, the difference between infested and uninfested dogs was not significant.

With respect to helminth and protozoan parasites found in collected feces, a negative association was found between CVL prevalence in sites where protozoan cysts and oocysts were present (z = 7.677, p = 0.009). Of these sites, only one was positive for the detection of antibodies in at least one dog. Association between canines with CVL and microfilarial infestation was not found in this study (p = 0.469).

Geospatial data was represented in a map by using colors to identify areas of different CVL prevalence (Fig 2). The result of global Moran´s I index was not significant in both sampling years (2014: z = -0.71, p = 0.477; 2018: z = 0.944, p = 0.344), meaning that there was not enough evidence to reject the randomness null hypothesis and that the distribution of the global CVL cases in PI would then respond to spatial randomness. Nevertheless, when performing local analysis of clustering, six sites in 2014 and one site in 2018 presented significant autocorrelation with neighboring sites (Fig 2). The first sampling year, four sites were classified in the H/L quadrant of Moran Scatterplot, one in the L/H quadrant and one in the H/H quadrant, while in 2018, there was a site belonging to the L/L quadrant (Table 3).

**Table 3:** Moran´s I local autocorrelation value and significance level of sites showing spatial autocorrelation with neighboring sites in Puerto Iguazú city in years 2014 and 2018.

Sampling year	Site (North to South)	Moran Scatterplot quadrant	Z-score	P-value
2014	1	H/L	-2.34	0.019
	2	L/H	-2.35	0.018
	3	H/H	2.79	0.005
	4	H/L	-3.37	<0.001
	5	H/L	-2.9	0.036
	6	H/L	-1.98	0.047
2018	1	L/L	1.98	0.046

H, sites with high standardized prevalence values; L, sites with low standardized prevalence values.

## Discussion

Differences in CVL prevalence were significant between sampling areas, being higher in PI city, the largest and most urbanized area. Although there is a lack of knowledge on dog population in these areas, possibly the population density is higher in PI, which implies that CVL transmission intensity in the other areas could be lower [34]. Besides, CVL transmission in the South Cone of South America is predominantly urban [9], and the vector *Lu*. *longipalpis* was found in PI since 2010 [12], but not in PL or the other sampled sites up to 2014. Further, as it was observed also in the Brazilian side, the border cities of Brazil and Argentina, Foz do Iguaçu and Puerto Iguazú respectively, seem to be a 'gateway for CVL’ spread to the western portion of the state of Paraná-Brazil [8] and northern region of Misiones-Argentina, providing allochthonous CVL cases. Therefore, in order to forecast the spatial spread of urban CVL and so HVL, cities prevalence, road network, transit and migration fluxes should be addressed [35,36].

In this study, CVL seroprevalence decreased in PI city in a four year period, while during this period two cases of HVL were reported from the city, both in 2014 [20]. Neither dog nor phlebotomine systematic control measures were performed, but several factors may contribute to decrease this prevalence: information on the disease delivered by researchers, local government and non-government organizations, removing of positive dogs by owners, and repeated fumigations for dengue transmission control due to an epidemic with a focus in PI in 2016 [37]. However, the significant decrease in CVL prevalence from 26.18% to 17.50% without further HVL cases after the initial emergence may be also associated to a transmission stabilization trend in dog population. A study conducted during years 2011–2012 in PI showed higher abundances of *Lu*. *longipalpis* from spring to autumn, with capture peaks in autumn [23]. High *Lu*. *longipalpis* abundances were recorded even during winter, so vector-host contact could be happening throughout the year [23]. On the other hand, CVL is a parasitic disease that produces chronic infections with a very unspecific incubation period. That is, since the vector inoculates the parasites, they can enter and survive long periods in macrophages, evading its immune system [38]. Even in times of lower vector abundance, horizontal and vertical transmission of *L*. *infantum* between dogs should not be underestimated, since these have not been compared with vector transmission. In this context, it might be possible to dismiss seasonality as a determining factor affecting seroprevalence in PI. Nevertheless, an effect of the environmental conditions given by the different sampling moments is not discarded, but a longitudinal study is necessary to evaluate this. On the other hand, to sample clusters of five dogs around a site prone to have vectors (to increase vector trapping sensitivity) could bias the dog prevalence to higher values than those obtained by an eventual random sampling [13], but the CVL prevalence obtained for PI is of the same order as those obtained in emergent cities of Brazil as the closest one to PI, Foz do Iguaçu [8,39].

Regarding 2014 dog variables model, dog age and no use of repellent were positively associated to CVL seropositive dogs. Prevalence was higher in adult and old dogs (> 1 year old), in agreement with other authors [40,41]. The age of the dogs could be related to the cumulative time elapsed of exposure to sandfly bites and the habit of sleeping outside the home, more frequent in adult dogs. In other studies, dogs staying in the backyard were associated with *L*. *infantum* infection, as well as the number of dogs living in the same house [42,43]. In this study, the age of the dogs and the habit of sleeping outside the houses showed a significant association (F = 19.625, p<0.001); 6.44% of all sampled dogs slept indoor but this proportion rose to 21.43% when only puppies were considered. Repellent application to dogs was extremely low in all sampled areas, which is an indicator of failure of control campaign messages and responsible ownership conducts, and lack of affordability of repellents by vulnerable families [44]. Distribution of CVL did not show significant associations with dog variables in 2018. This result may be due to the size of the sub-sampling together with a lower prevalence related to the stabilization process (see below spatial analysis discussion).

Differences in clinical signs model outputs between years 2014 and 2018 are reflected in raw data. In the first sampling year there was a higher proportion of positive dogs with clinical signs and a higher rate of infection by RDT, which has greater sensitivity in symptomatic dogs [45,46]. In 2018 a higher number of dogs had good body condition, lesser had ophthalmological and dermal signs, there were more active dogs and fewer cases of adenomegaly and onycogryphosis. In this second sampling period, dermal and ophthalmological signs and onycogryphosis were no longer associated with CVL seropositive dogs; instead, adenomegaly became important in sign manifestations. Adenomegaly is a hard detecting sign and was the predominant sign in oligosymptomatic dogs, contributing the most to positive cases in 2018, although results also show that it is a category of low certitude for diagnosis, prone to have more subjective bias, and that signs could be caused by many other pathologies different than CVL. However, even if some asymptomatic animals in 2018 were not detected by RDT due to lower parasite loads and test threshold, the changes in clinical signs between both periods could be due again to a stabilization trend of the transmission and death of polysymptomatic dogs, segregating more clearly the categories of non-reactive, reactive olygosymptomatic almost equivalent to asymptomatic, and polysymptomatic dogs. Further, not all seropositive dogs had positive lymph node smears and/or PCR, which could be due to low parasite loads in seropositive dogs, leading to a decrease in these tests sensitivity [47,48]

Ectoparasite loads were similar to those of other studies in the area [49,50]. Transmission of *L*. *infantum* by ectoparasites was proposed by other authors [49,51], but vectorial capacity of *R*. *sanguineus* and *C*. *felis* and its epidemiological role for CVL were not verified so far [52,53]. In this study, *R*. *sanguineus* presence was associated with anti-*Leishmania* antibodies as it was reported by Silva et al. (2018 [54]), but repellent could have an effect on tick control, since in 2014 there was a significant negative association between the use of repellent and the presence of ticks (z = -4,646, p = 0.0383). This year, the use of repellent was higher and the number of dogs infested with ectoparasites was lower than in 2018. Therefore, association between *Leishmania* and ticks is probably masked by repellent use, practice that is also an indicator of responsible pet ownership, socio-economical agency capacity and healthy environment. On the other hand, values of infestation could have been affected by ectoparasite population dynamics as the samplings were performed in different seasons each year.

Endoparasite loads were high in dogs, as seen in other studies in the area [55–57]. In turn, dog filarial loads were lower than those reported for the area [58,59]. High endoparasite loads are also often a result of inadequate pet sanitation due to lack of knowledge or lack of material resources. Just as owners cannot apply repellent to their pets and the environment, it is possible that those same owners do not treat their dogs against endoparasites. Nevertheless, a negative association between the occurrence of dogs with antibodies against *Leishmania* rK39 recombinant protein and the presence of other protozoa was found. Although environmental factors are modulators of host immune systems, many studies assess parasite interspecific competition as a strong driver of parasitism, as parasites can polarize the immune response [60,61]. For example, a strong Th1 response by *Toxoplasma* has been found to modulate and improve immune responses to *Leishmania* [60]. Following this example, intra-cellular protozoa as *Leishmania* sp. and intestinal protozoa both stimulate Th1 responses [62], suggesting seropositive dogs could have lower loads of other protozoa.

Regarding spatial analysis, areas of higher prevalence are randomly distributed on the first map, where they seem immersed in areas of lower prevalence (Fig 2A) as it was already described for *Lu*. *longipalpis* abundance [16]. In 2014 five high prevalence sites were surrounded by low CVL incidence (H/L). However, in 2018 there is a L/L point in a large area south of the city with low prevalence (Fig 2B). The change of autocorrelation prevalence spots distribution between years could be related with canine translocation within the city (approximately 50% of sampled dogs came from other neighborhoods) as the transit and traffic of dogs have not spatial contiguous patterns, and its effects increase with time since CVL emergence. So, CVL distribution could be explained not only by vector transmission as in new foci [8,63], but also by the structure of social networks of pets, including clusters of vertical and horizontal transmission between dogs. Therefore, there could be a lack of association of CVL with environmental variables related to vector transmission risk as Normalized Difference Vegetation Index (NDVI), distance to the nearest stream shore and presence of hens [7,64,65], while it is still associated with those environmental variables related to the socio-economic determination of urban visceral leishmaniasis [52,66,67]. In PI the areas of higher stable abundance of *Lu*. *longipalpis* up to 2012 did not overlap with the CVL distribution presented here [23], even after the progression of CVL incidence during this study. Therefore, in the city scenario in the scale studied, the spatial distribution of CVL seems to be a poor evidence for the spatial design of measures against the vector (environmental or chemical). On the other hand, CVL-HVL clusters could overlap despite the distribution of vector high abundance clusters due to the intensity of parasite circulation [5,21,66,68,69], although the lack of this association was also reported [70].

For control purposes, until we have better indicators of dog individual infectiousness to *Lu*. *longipalpis* the sensitivity of CVL diagnostic methods is still critical for control success, mainly due to low sensitivity of immunological tests in asymptomatic/oligosymptomatic dogs, that represent long-term parasite sources [68,71–73]. Therefore, for CVL control should be fostered responsible ownership to strength individual preventive measures like using repellents or collars [74,75], and anti-vectorial interventions in outdoor dog sleeping sites (indoor sleeping sites entail other risks related to parasites and vectors). However, for collective health and population-based measures to reduce both CVL and HVL, political will and adequate resources are required for successful dog castration programs, law regulation for pet breeding-traffic to reduce vertical transmission and stray dog burden, laboratory-based surveillance with quality-control reference, and even equitable access to preventive strategies.

Historically, once urban transmission of visceral leishmaniasis with vector, and autochthonous VCL and HCL cases was reported for the first time in Argentina, some of the authors belonging to the Leishmaniasis National Program recommended the culling of seropositive dogs as part of the integrated control management. This recommendation was based on the pattern of cases along time, the HCL/VCL proportion and fatality rate in some cities of Brazil during the recent spread of urban American visceral leishmaniasis [21,76–79]. The goal of this intervention, the removal of active reservoirs, in Argentina as in other countries was ineffectively operationalized due to low sensitivity of diagnostic tests without identification of actual core-transmitters, time elapsed between diagnosis and culling, quick replacement of culled dogs by high susceptible puppies without changes in the environmental risk factors, and rejection of the population mainly regarding to infected asymptomatic dogs. However, despite the high CVL prevalence and this conflictive scenario, progression of CVL in Argentina seems to stabilize, as observed in this study, with HVL scattered in space and time [20]. Therefore, new recommendations are required for this scenario while agreements between programs, veterinarians of private practice and the community are achieved.

In conclusion, in order to develop effective and feasible strategies for CVL and HVL prevention and control in these southern scenarios, modeling will be required to identify factors that drive disease stabilization/destabilization, assessing from biological (parasite-vector-host characteristics, seasonal dynamics) to socio-economical determinants and cultural and political practices. Disease stabilization is a result of an equilibrium trend process in a community due to its resistance or resilience after a perturbation as a pathogen emergence [80–83]. However, as the equilibrium is no a static state, the eventual inputs that could change the pattern should be evaluated, mainly the massive movement of infected-susceptible dogs and humans (migration, dog breeding), time-space peaks of vector abundance due to climatic events or environmental changes, unsystematic culling of dogs with chronic infection and immediate replacement with susceptible dogs, intervention in vector ‘hot spots’, CVL ‘hot spots’ and around sites of transmission of HVL cases, and cost-effectiveness of measures proposed for high endemic areas [79]. On the other hand, when a locality initially detects native cases of CVL, still associated in space with vector distribution, the vector-reservoir focal control is feasible together with sensitization of the health system to look for CVL and HVL cases, and settlement of programs that include the surveillance of CVL or vector spread.
